# TLR4-mediated brain inflammation halts neurogenesis: impact of hormonal replacement therapy

**DOI:** 10.3389/fncel.2014.00146

**Published:** 2014-05-27

**Authors:** Abdeslam Mouihate

**Affiliations:** Department of Physiology, Faculty of Medicine, Health Sciences Centre, Kuwait UniversitySafat, Kuwait

**Keywords:** microglia, TNF-α, COX-2, doublecortin, NFκB, neuroprotection

## Abstract

Experimental and epidemiological data show that the severity and the duration of brain inflammation are attenuated in females compared to males. This attenuated brain inflammation is ascribed to 17β-estradiol. However, several studies suggest that 17β-estradiol is also endowed with proinflammatory properties. The aim of the present study is to assess the effect of hormonal replacement therapies on lipopolysaccharide (LPS)-induced brain inflammation and its consequent effect on newly born neurons. Bilaterally ovariectomized rats received intrastriatal injection of LPS (250 ng/μl) and were subsequently given daily subcutaneous injections of either vehicle, 17β-estradiol (25 μg/kg) or 17β-estradiol and progesterone (5 mg/kg). Microglial activation and newly born neurons in the rostral migratory stream were monitored using double immunofluorescence. Nuclear factor κB (NFκB) signaling pathway and its target inflammatory proteins were assessed by either western blot [cyclooxygenase-2 (COX-2) and interleukin-6 (IL-6)] or enzyme-linked immunosorbent assay [tumor necrosis factor-α (TNF-α)]. LPS-induced activation of microglia, promoted NFκB signaling pathway and enhanced the production of proinflammatory proteins (TNF-α and COX-2). These proinflammatory responses were not attenuated by 17β-estradiol injection. Supplementation of 17β-estradiol with progesterone significantly dampened these proinflammatory processes. Interestingly, LPS-induced brain inflammation dampened the number of newly born neurons in the rostral migratory stream. Administration of combined 17β-estradiol and progesterone resulted in a significantly higher number of newly born neurons when compared to those seen in rats given either vehicle or 17β-estradiol alone. These data strongly suggest that combined 17β-estradiol and progesterone, and not 17β-estradiol alone, rescues neurogenesis from the deleterious effect of brain inflammation likely via the inhibition of the signaling pathways leading to the activation of proinflammatory genes.

## INTRODUCTION

Brain inflammation is a common symptom that develops as a result of many infectious diseases (e.g., *E.coli* meningitis, HIV encephalopathy, West Nile virus induced dementia; Nau and Bruck, 2002; Gendelman and Persidsky, 2005; Hayes et al., 2005), neurological diseases (Eikelenboom et al., 2002; Streit, 2004; Nagatsu and Sawada, 2005; Stys et al., 2012), stroke and brain trauma (Spencer et al., 2008; Lambertsen et al., 2012). While moderate brain inflammation plays an important role in the repair process following an insult, prolonged and exacerbated brain inflammation hampers neuronal survival and inhibits neuronal renewal (neurogenesis; Liu et al., 2001; Springer et al., 2001; Ekdahl et al., 2003; Monje et al., 2003; Bessis et al., 2007) and consequently negatively impacts brain integrity and function.

Experimentally, a well-established model of brain inflammation consists of the local application of lipopolysaccharide (LPS; the outer coat of Gram negative bacteria) within the brain parenchyma, at the level of the striatum (Nadeau and Rivest, 2002; Cunningham et al., 2005; Soucy et al., 2005; Glezer et al., 2007; Hunter et al., 2007). Once administered, LPS binds to a specific receptor called toll like receptor 4 (TLR4) expressed largely on microglia; the immune competent cells within the brain (Laflamme and Rivest, 2001; Lehnardt et al., 2002, 2003). TLR4 activation results in the phosphorylation of a series of intracellular kinases culminating in the phosphorylation of an inhibitory factor called IκB. The phosphorylation of IκB results in the release of a nuclear transcription factor: nuclear factor κB (NFκB; Rivest, 2003; Dev et al., 2011), which translocates into the nucleus and induces the transcription of inflammatory genes, such as cyclooxygenase-2 (COX-2), tumor necrosis factor-α (TNF-α), interleukin-1β (IL-1β), and interleukin-6 (IL-6) (Libermann and Baltimore, 1990; Cao et al., 1997; Rivest, 2003; Krakauer, 2004; Chew et al., 2006; Brasier, 2010).

In addition to these transcriptional events, microglia adopt morphological changes to allow for motility and secretory functions (Dheen et al., 2007). Indeed, in non-pathological conditions, microglial cells adopt a resting shape characterized by a small perikarya and numerous and long processes (Spencer et al., 2008; Ousman and Kubes, 2012). Once activated by bacterial LPS, microglial cells morph into an ameboid shape with fewer and shorter processes, increase in number (mitosis) and mount local inflammatory responses (Kim et al., 2000; Nadeau and Rivest, 2002). Exacerbated microglial activation and prolonged production of inflammatory molecules creates hostile environment for both neuronal survival (Springer et al., 2001; Cunningham et al., 2005) and neurogenesis (Ekdahl et al., 2003; Monje et al., 2003).

Epidemiological and experimental data strongly suggest that the severity and duration of brain inflammation is higher in males compared to females (Roof and Hall, 2000; Murray et al., 2003). It had been advanced that this dampened brain inflammatory response in females is brought about by the anti-inflammatory role of female sex hormones (namely 17β-estradiol [E] and progesterone [Pr]; Stein and Hoffman, 2003; Amantea et al., 2005). However, the potential beneficial role of ovarian hormones remains highly debated and controversial as these hormones have been shown to either promote (Calippe et al., 2008, 2010; Rettew et al., 2009; Seillet et al., 2012) or suppress brain inflammatory responses (Pozzi et al., 2006; Vegeto et al., 2008).

We have previously shown that a hormonal replacement therapy (HRT) that combines E and Pr dampens neuroimmune responses to systemic inflammation in ovariectomized (OVX) rats (Mouihate and Pittman, 2003). Conversely, an HRT based on E alone was required for promoting LPS-induced brain inflammatory response (Soucy et al., 2005). Thus, in the present paper, we hypothesize that an HRT based on E and Pr, but not that consisting of E alone, will likely dampen brain inflammatory response. To this aim we assessed the effect of the two clinically prescribed HRT regimens (e.g., E + Pr, or E alone) on microglial activation, and the molecular events leading to the inflammatory response including the activation of NFκB signaling pathways, proinflammatory cytokines and COX-2. Because brain inflammation dampens neurogenesis and that the striatal inflammatory response spreads to the rostral migratory stream (RMS), a route of newly born neurons migrating toward the olfactory bulb (Lepousez et al., 2013), we explored whether the HRT impact on brain inflammation is associated with survival of newly born neurons in the RMS.

## MATERIALS AND METHODS

Female Sprague Dawley rats weighting 250–270 g were bred in the Animal Resources Centre at the Health Sciences Centre, Kuwait University. The room temperature was set to 22°C and the rats lived under a 12 h light/dark cycle (7 a.m.–7 p.m.). They were pair-housed, and had access to pellet chow and water *ad libitum*. All experiments were done in accordance with guidelines on humane handling of experimental animals as established by the Canadian Council on Animal Care. The procedures employed were approved by the Animal Resources Centre of Kuwait University. All efforts were made to minimize animal suffering.

### ANIMAL SURGERIES

Female rats (250–270 g) were anesthesized with an i.p. injection of a mixture (1 ml/kg b.w.) of ketamine (50 mg/ml) and xylazine (3 mg/ml) and both ovaries were surgically removed. The OVX rats were then left undisturbed for two weeks to allow the clearance of circulating ovarian hormones as previously described (Mouihate and Pittman, 2003). On day 15 post-ovariectomy, anesthetized (mixture of ketamine–xylazine) OVX rats were positioned in a stereotaxic apparatus (387673937Harvard Apparatus, Holliston, MA, USA) to receive an intracerebral injection of LPS using a 10 μl Hamilton syringe (Hamilton Bonaduz, GR, Switzerland, 32 ga). The syringe was guided stereotaxically to the level of the striatum with the following coordinates relative to the bregma: anterior/posterior, +1.0 mm; lateral, 2.5 mm; ventral, –4.5 mm and 2 μl of LPS solution (250 ng/μl solution) was infused for a period of 2 min. After LPS injection, the syringe was left in place for an extra 3 min to allow for complete infusion as previously described (Kim et al., 2000; Nadeau and Rivest, 2002).

OVX rats received an HRT consisting of daily s.c. injection of either E (25 μg/kg, 1,3,5,10-estratrien-3,17β-diol3-benzoate, Sigma Aldrich, St. Louis, MO, USA) alone or combined E (25 μg/kg) and Pr (5 mg/kg, 4-pregnene-30,20-dione, Sigma, St. Louis, MO, USA) dissolved in sesame oil. Control animals received s.c. injection of an equivalent volume of sesame oil. The initial HRT injection started at 2 h post intra-cerebral administration of LPS. The doses of injected ovarian hormones are within physiological ranges as was previously described (Boling and Blandau, 1939; Mouihate et al., 1998; Mouihate and Pittman, 2003).

### IMMUNOFLUORESCENCE

On the third day post LPS injection at 10–12 a.m. [day 3 corresponds to the peak of brain inflammation (Soucy et al., 2005)], rats were transcardially perfused with phosphate buffered saline (PBS) solution (NaCl, 137 mM; KCl, 2.7 mM; Na_2_HPO_4_, 10 mM; KH_2_PO_4_, 1.8 mM) followed by fixative (10% neutral formalin). Rat brains were post-fixed overnight, embedded in paraffin and processed for immunofluorescence. Paraffin embedded brains were cut at the level of the striatum (5 μm, microtome) and mounted on superfrost plus slides (VWR, Arlington Heights, IL, USA). Hydrated brain sections were exposed to a primary Iba-1 antibody (ionized calcium binding adapter molecule 1; a microglial marker) made in rabbit (overnight at room temperature, 1:1000; Wako Chemicals USA, Inc., Richmond, VA, USA), followed by a secondary antibody (2 h, 1:1000; donkey anti-rabbit IgG (Alexa Fluor 488); Life Technologies, Carlsbad, CA, USA) as was previously described (Spencer et al., 2008). To detect newly born neurons, brain sections were incubated in doublecortin antibody made in goat (overnight at room temperature 1:1000, Santa Cruz Biotechnology, Santa Cruz, CA, USA) followed by a secondary antibody [2 h; donkey anti-goat IgG (Alexa Fluor 555); Life Technologies, Carlsbad, CA, USA]. Doublecortin expression was used for monitoring ongoing neurogenesis (Rao and Shetty, 2004; Couillard-Despres et al., 2005). Labeled brain sections were viewed using a confocal laser scanning microscope (Carl Zeiss Microscopy GmbH). Slides were re-coded by a laboratory member not involved in doublecortin and microglial counting to allow for blind observation and counting of microglial cells at the site of LPS injection and doublecortin in the RMS. Activated and non-activated microglia were observed under 40× objective, counted by an experimenter blind to the rats’ treatment group and evaluated as previously described (Spencer et al., 2008). In brief, microglial cells which have small perikarya and long thin branches were classified as rested, while those showing large perikarya and short and relatively think processes were considered active microglia (Spencer et al., 2008).

Microglial cells and doublecortin containing cells were counted from nine different sections at 20 μm apart from each other. Doublecortin containing cells in the RMS were viewed under a 40× objective and counted. The total of doublecortin containing cells is presented. From each of the nine sections, three visual fields below the site of LPS injection were taken under a 40× objective and were used for the microglial count. The microglial images were viewed using ImageJ software (version 1.44) developed at the National Institute of Health (USA; Schneider et al., 2012), and the cells were counted using a cell counter macro in ImageJ. The data are presented as the average of number of microglia/counting area.

### ENZYME-LINKED IMMUNOSORBENT ASSAY

In a separate series of experiments, new group of rats was OVX and received HRT treatment as described above. The OVX rats were transcardially perfused with PBS and ~1 mm^3^ brain tissue at the site of LPS injection were collected as fresh tissue, snap frozen in liquid nitrogen and stored in deep freezer (–80°C) until used for either western blot or ELISA. TNF-α levels were assayed using a specific rat ELISA kit (Life Technologies, Carlsbad, CA, USA). The minimum detectable concentration is 4 pg/ml. The inter-assay variability is 7.8–9% CV and the intra-assay variability, 4.3–6.9% CV. All samples were assayed in duplicate and representatives from all groups were analyzed in the same assay.

### WESTERN BLOT

Due to the small amount of brain tissue obtained from each animal (~300 μl of protein solution), we were not able to perform multiple ELISAs for different proinflammatory cytokines. We took advantage of the availability of an IL-6 antibody suitable for western blot analysis to explore the impact of HRT on IL-6 expression in LPS-induced brain inflammation. A different series of western blot were performed on the same protein extracts to monitor the expression of the phosphorylated levels of IκB (p-IκB), an indicator of activation levels of NFκB signaling pathway. Proteins (60 μg per well) were separated by 12% SDS PAGE, transferred to a nitrocellulose membrane, and incubated overnight at 4°C with primary antibodies to either IL-6 (1:1000, goat antibody from R&D Systems, Minneapolis, MN, USA), COX-2 (1:2000; rabbit antibody from Cayman Chemical, Ann Arbor, MI, USA), or p-IκB (1:2000; mouse antibody from Cell Signaling Technology, Beverly, MA, USA). After washing, the membranes were incubated for 2 h at room temperature with horseradish-peroxidase conjugated secondary antibodies (donkey anti-goat for IL-6, donkey anti-rabbit for COX-2, or donkey anti-mouse for p-Iκ at a dilution of 1:2000; Santa Cruz Biotechnology, Santa Cruz, CA, USA). Protein bands were detected after application of chemiluminescence substrate (ECL plus kit; GE Healthcare) and exposure to Kodak X-Omat film (Eastman Kodak). The nitrocellulose membranes were subsequently stripped with β-mercaptoethanol (Sigma-Aldrich, St. Louis, MO, USA) and reused to detect the housekeeping protein actin (1:5000, rabbit antibody from Sigma Aldrich, St. Louis, MO, USA) or total IκB (t-IκB; 1:2000, rabbit antibody from Santa Cruz Biotechnology, Santa Cruz, CA, USA). The membranes were subsequently incubated for 2 h at room temperature with horseradish-peroxidase conjugated secondary donkey anti-rabbit (1:2000, Santa Cruz Biotechnology, Santa Cruz, CA, USA) and protein bands were detected as previously described (Mouihate et al., 2005; Mouihate et al., 2010).

### DATA ANALYSIS

For western blot analysis, densitometric analysis was performed as previously described (Mouihate et al., 2006, 2010). The ratios of optical density values of COX-2/actin, IL-6/actin or p-IκB/t-IκB were calculated and expressed as a multiple of the values in control animals that received vehicle. Western blot data, TNF-α (ELISA) levels and doublecortin containing cells (immunohistochemistry) were compared using one way ANOVA followed by Student–Newman–Keuls *post hoc* comparisons (for three treatment groups (O, E and E + Pr). The number of doublecortin in ipsilateral and contralateral sides to LPS injection was compared using Student’s *t*-test. Counts of activated and resting microglia were compared using two way ANOVA followed by Student–Newman–Keuls *post hoc* comparisons. The significance was accepted at *p* < 0.05.

## RESULTS

**FIGURE 1:**
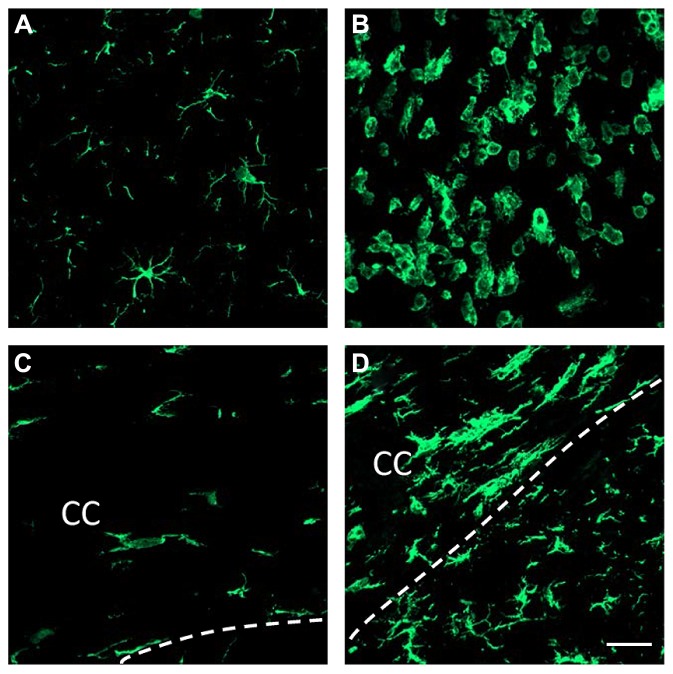
**LPS-induced brain inflammation.** Immunofluorescent detection of brain microglia using Iba-1 antibody 3 days after the injection of either saline **(A,C)** or LPS **(B,D)** into the striatum. Microglia show “resting” features in the contralateral side to the LPS injection **(A)**. Microglial cells have long processes and small cell bodies. In contrast, microglial cells at the site of injection of LPS show active features whereby their cell bodies enlarge, their processes retract and their number increases **(B)**. LPS injected into the striatum also activates microglia within the *corpus callosum* (CC; **D**). Dashed lines delineate the lower limit of the *corpus callosum*. Note the microglial alignment with the white matter axonal tracts **(C,D)**. Scale bar = 20 μm.

**Figure 1** shows resting microglia in the contralateral side to the LPS injection and activated microglia in the ipsilateral side of LPS injection. Microglia in the contralateral side to the LPS injection elicit features of resting state characterized by small perikarya and thin branches in both gray matter (**Figure 1A**) and white matter such as the corpus callosum (**Figure 1C**). Intrastriatal injection of LPS led to a drastic change in both the cell number and shape of microglial cells in the ipsilateral side (**Figure 1B**). This inflammatory response spreads to the corpus callosum, where microglial cells show large perikarya and small branches (**Figure 1D**).

**FIGURE 2:**
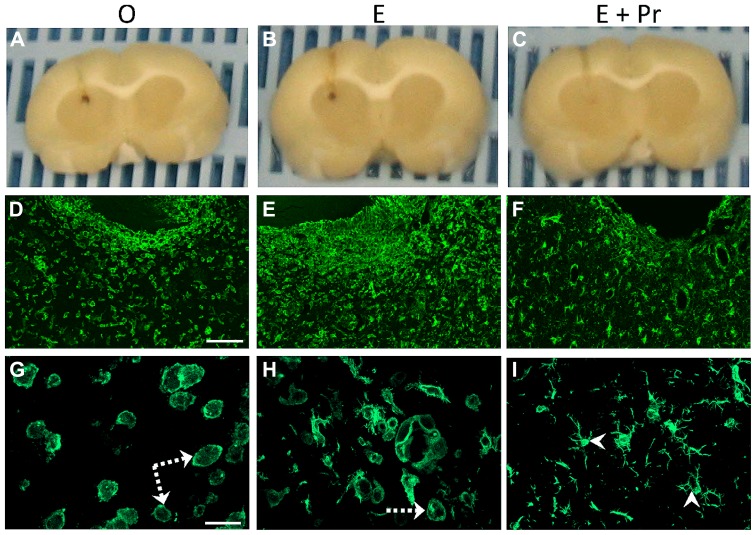
**Hormonal replacement therapy based on combined estradiol and progesterone dampens microglial activation.** Brain inflammation was induced by intracerebral injection of LPS to OVX rats given either vehicle (O), estradiol (E) or combined estradiol and progesterone (E + Pr) treatment. Formalin fixed brains **(A–C)** show the location of LPS injection. Intracerebral injection of LPS to vehicle treated rats (O) induces an increased number of microglial cells expressing Iba1 **(D,G)**. These microglial cells show a round shape with small processes (dashed arrows). Injection of E alone to OVX rats did not affect microglial activation **(E,H)**, while the microglial cells in OVX rats given E + Pr treatment **(F,I)** show signs of resting state with elongated processes (arrowheads) and relatively smaller perikarya. Scale bar: 100 μm in **D–F** and 20 μm in **G–I**.

**FIGURE 3:**
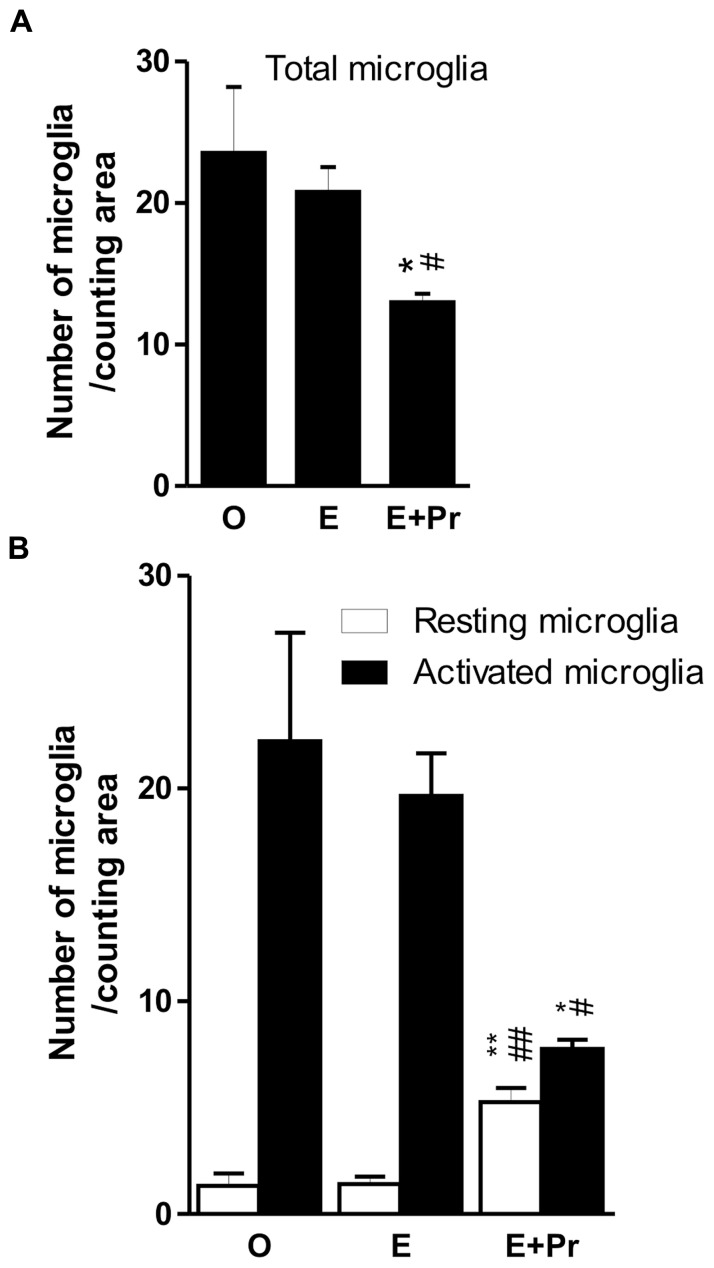
**Combined estradiol and progesterone injections halts microglial activation.** The total number of microglia was high in inflamed rat brains of OVX rats given either vehicle (O) or estradiol (E). Injection of both E and progesterone (E + Pr) significantly reduced the number of microglia **(A)**. The number of resting microglia was very low in the inflamed brain area of OVX rats given either vehicle or estradiol **(B)**. This low number of resting microglia was mirrored by an increased number of activated microgilal cells. The number of resting microglia increased while that of activated ones decreased in E + Pr treated rats. *E + Pr *vs*. O groups, ^#^E + Pr *vs*. E groups. *^,#^*p* < 0.05, **^,##^*p* < 0.01.

In order to determine whether HRT regimens affect LPS-induced brain inflammation, microglial activation was assessed in the inflamed striatal area of OVX rats given either the HRT regimens or vehicle. As can be seen in **Figure 2**, LPS promoted strong microglial activation in vehicle-treated OVX rats (**Figure 2**/left column, [O]). This microglial activation was not affected by an HRT consisting of 17β-estradiol alone (**Figure 2**/middle column, [E]). However, when OVX rats were given an HRT containing both 17β-estradiol and progesterone (E + Pr), microglia showed features of resting state (**Figure 2**/right column, [E + Pr]). **Figures 3A, B** show that the numbers of total microglia and the activated microglia were high in vehicle treated OVX rats. Such numbers were not significantly affected by E treatment [E (*n* = 5) *vs*. O (*n* = 4) rat groups, *p* > 0.05] but were significantly reduced in the brain of OVX rats given E + Pr treatment [E + Pr (*n* = 5) *vs*. O (*n* = 4) rat groups, *p* < 0.05]. Conversely, E + Pr treatment resulted in a significant increase in the number of rested microglia [**Figure 3B**; E + Pr (*n* = 5) *vs*. O (*n* = 4) rat groups, *p* < 0.01].

**FIGURE 4:**
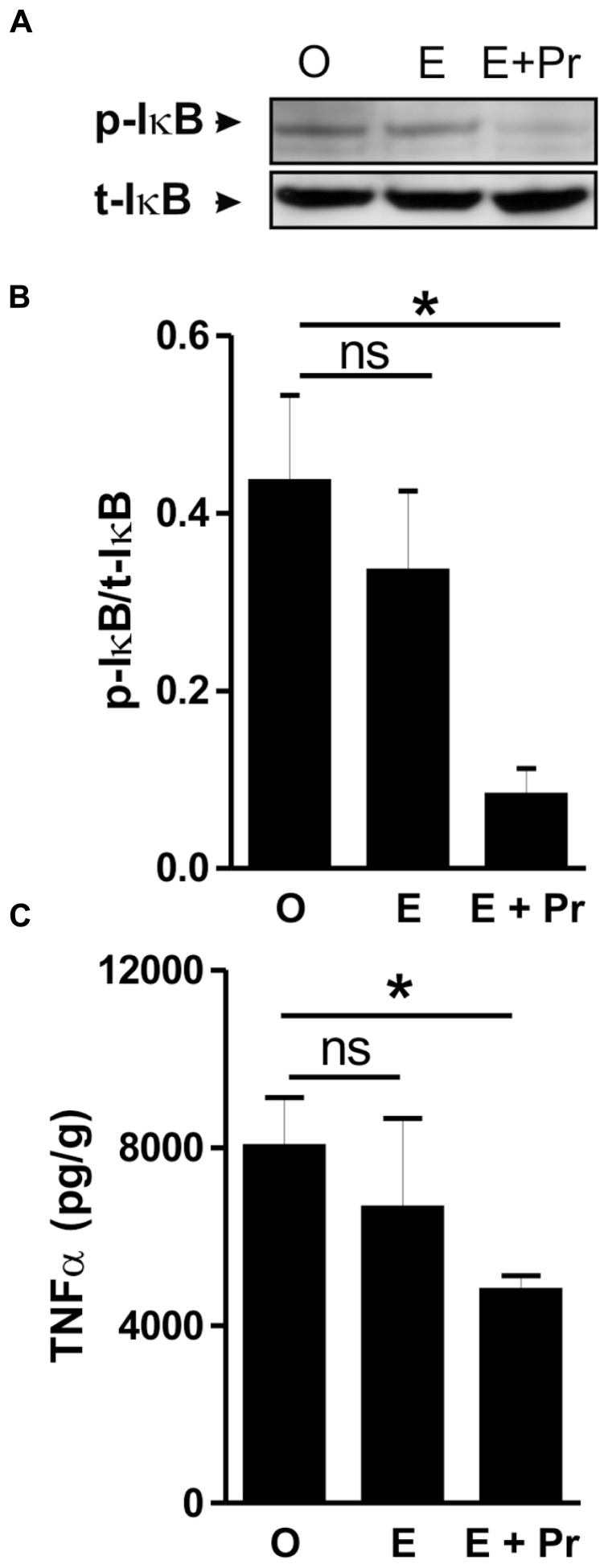
**Combined estradiol and progesterone injections suppress NFκB signaling and TNF-α production.** The panel in **A** shows a micrograph of a western blot detection of the inhibitory κB (t-IκB) and its phosphorylated form (p-IκB) in the inflamed area of the brain of OVX rats given either vehicle (O), estradiol (E) or E and progesterone (E + Pr). The levels of p-IκB were not affected by estradiol treatment. Densitometric analysis **(B)** shows that the levels of p-IκB were significantly reduced in E + Pr rat group. The ELISA measurement of TNF-α levels in the inflamed brain is shown in **C**. The levels of TNF-α observed in control group (O) were not affected by E treatment but were significantly reduced in the brains of E + Pr rat group. *p < 0.05, ns = not significant.

**FIGURE 5:**
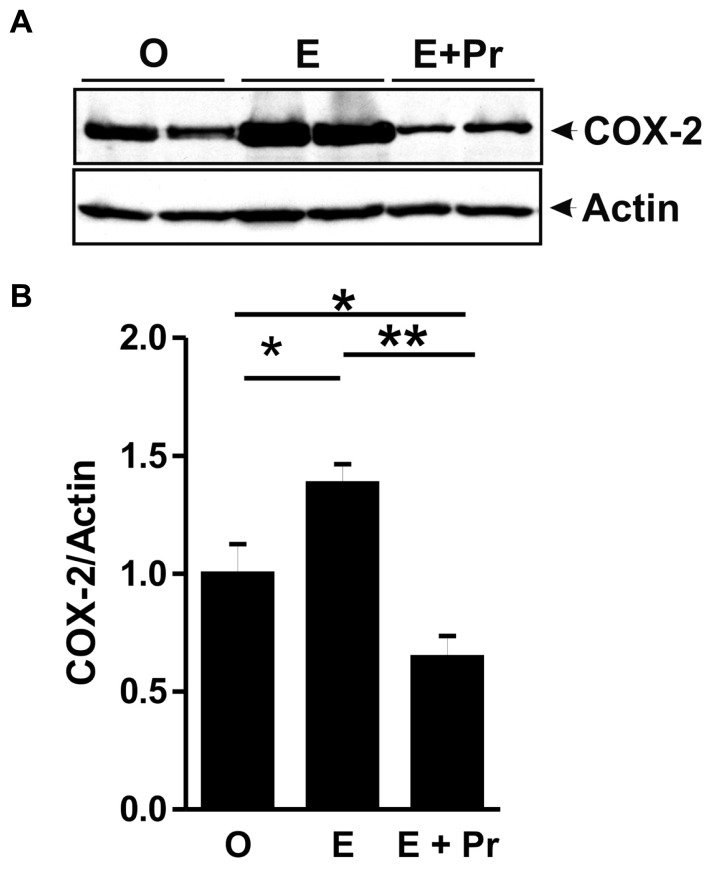
**Combined estradiol and progesterone injections dampen COX-2 expression.** The panel in **A** shows a micrograph of a western blot detection of COX-2 in the inflamed area of the brain of OVX rats given either vehicle (O), estradiol (E) or E and progesterone (E + Pr). Densitometric analysis **(B)** shows that the levels of COX-2 were significantly enhanced by estradiol treatment (E) when compared to control group (O). On the other hand, E + Pr treatment dampened COX-2 expression (E + Pr). **p < 0.01, *p < 0.05.

**FIGURE 6:**
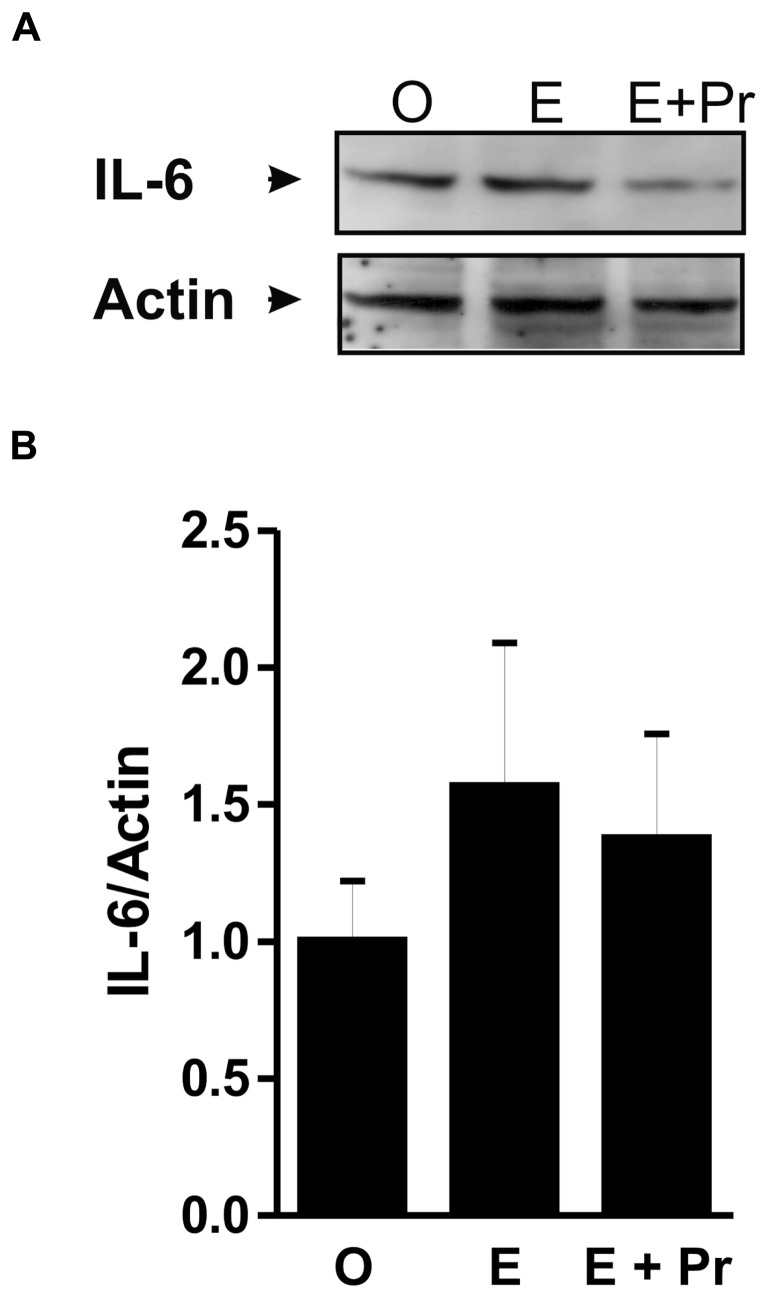
**HRT does not affect the production of IL-6 protein.** The panel in **A** shows a micrograph of a western blot detection of IL-6 protein in the inflamed area of the brain of OVX rats given either vehicle (O), estradiol (E) or E and progesterone (E + Pr). The panel in **B** shows densitometric analysis of the western blot. The levels of IL-6 were slightly but not significantly increased in the E rat group when compared to O rat group. These IL-6 levels were not affected by E + Pr treatment (*p* > 0.05).

Once activated, microglial cells start to synthesize a set of proinflammatory cytokines under the control of NFκB signaling pathway (Ransohoff and Brown, 2012). To test whether HRT effect on brain inflammatory response is associated with alteration in the NFκB signaling pathway, we semi-quantified *p*-IκB as an index of the activity of NFκB (Ellis et al., 2005; Mouihate et al., 2006; Hayden and Ghosh, 2012). As can be seen in **Figure 4A**, there was a detectable amount of p-IκB in the striatal region injected with LPS in the vehicle-treated OVX rats (O). Densitometric analysis (**Figure 4B**) showed that these p-IκB levels were not significantly reduced in E-treated OVX rats treatment [E (*n* = 5) *vs*. O (*n* = 5) rat groups, *p* > 0.05]. An HRT containing both E and Pr resulted in a significant reduction in the levels of p-IκB [E + Pr (*n* = 5) *vs*. O (*n* = 5) rat groups, *p* < 0.05]. Once activated, the NFκB signaling pathway leads to the production of proinflammatory cytokines, chief among which is the TNF-α (Frei et al., 1987). As can be seen in **Figure 4C**, the levels of TNF-α in the LPS-injected striatal region of OVX rats given vehicle treatment were not significantly affected in E-treated OVX rats [E (*n* = 5) *vs*. O (*n* = 5) rat groups, *p* > 0.05]. However, TNF-α levels were significantly reduced in the LPS-injected striatum of OVX rats given E + Pr treatment [E + Pr (*n* = 5) *vs*. O (*n* = 5) rat groups, *p* < 0.05]. In addition to TNF-α, *COX-2* represents another important inflammatory gene activated through the NFκB signaling pathway (Nadjar et al., 2005). Thus the impact of HRT regimens on COX-2 protein expression in the inflamed brain was assessed. Immunoblot and densitometric analysis in **Figure 5** show that the COX-2 protein expression was enhanced when OVX rats received an HRT regimen consisting of E alone [E (*n* = 5) *vs*. O (*n* = 5) rat groups, *p* < 0.05] but was significantly attenuated when both E and Pr were administered [E + Pr (*n* = 5) *vs*. O (*n* = 5) rat groups, *p* < 0.05]. Surprisingly, none of the HRT regimens significantly altered the levels of IL-6 (**Figure 6**), a proinflammatory cytokine which is also under the control of the transcriptional effect of NFκB.

**FIGURE 7:**
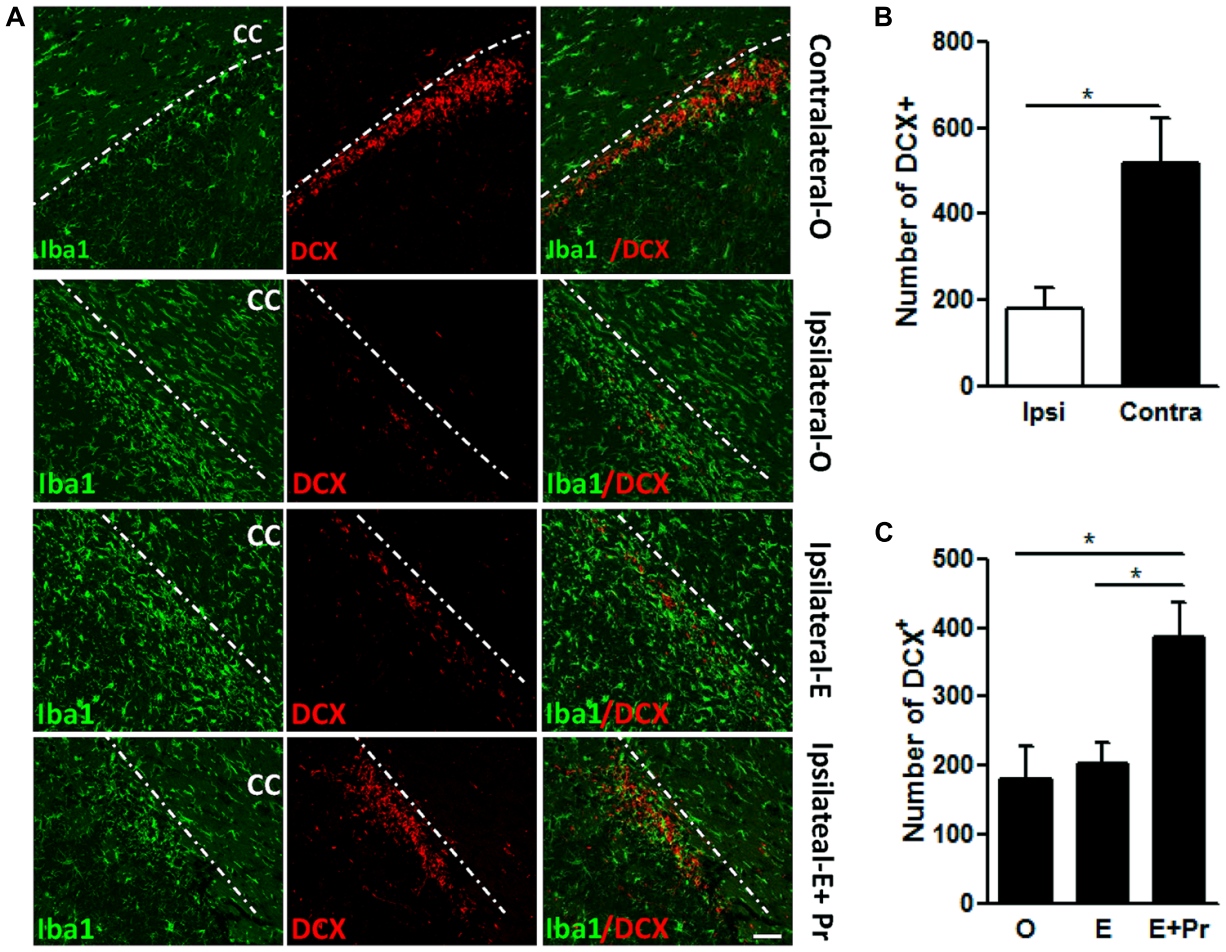
**Combined estradiol and progesterone injections rescue neurogenesis in the rostral migratory stream.** In **A**, rat brains were subjected to a double immunostaining for microglial marker (Iba-1, green) and a marker of newly born neurons (DCX, red). Microglial cells adopt a resting state in the contralateral side to LPS injection site (Contralateral-O). In the ipsilateral side to the LPS injection site, brain inflammation reduced the number of DCX containing cells in OVX rats given oil treatment (Ipsilateral-O and the graph bar in **B**). Estradiol did impact neither LPS-induced inflammation nor LPS-induced reduction in newly born neurons (Ipsilateral-E). Brain inflammation was reduced while the number of newly born neurons was higher in the brain of OVX rats given combined injection of estradiol and progesterone (Ipsilateral-E + Pr and the graph bar **C**). This immunostaining micrograph is representative of 3–6 different animals in each treatment group. Dashed line denotes the demarcation between the RMS and corpus callosum (CC). Scale bar = 50 μm; *p < 0.05.

Evidence strongly suggests that brain inflammation that accompanies many neurodegenerative diseases can negatively impact neuronal survival (Ekdahl, 2012). In the present study, we assessed the impact of the brain inflammatory response to LPS on the density of newly born neurons. The inflammatory response to intra-striatal injection of LPS spreads to areas known for the migration of newly born neurons in the RMS. As can be seen in **Figure 7**, the spreading of inflammatory response resulted in a significant reduction in newly born neurons. We took advantage of this spreading inflammation to test whether the observed reduction in brain inflammation after E + Pr treatment is associated with the survival of newly born neurons. LPS-injected vehicle-treated OVX rats showed a strong inflammatory response which was associated with reduced number of DCX containing cells in RMS (**Figure 7**) when compared to the amount of DCX containing cells in the contralateral side to LPS injection (**Figure 7**, Ipsilateral-O *vs*. Contralateral-O and graph bar in B). Compared to oil injected rats (Ipsilateral-O), the amount of newly born neurons was higher in the RMS of OVX rats given E + Pr (**Figure 7**, Ipsilateral-E + Pr), but not in the RMS of OVX rats given E alone (**Figure 7**, Ipsilateral-E). The graph bar in **Figure 7C** shows that the number of DCX-containing cells is significantly higher in the RMS of E + Pr injected rats compared to those given oil or E alone.

## DISCUSSION

In the present paper, we have made several important and novel observations, (1) E + Pr but not E only based HRT significantly reduced LPS-induced microglial activation during brain inflammation in OVX rats, (2) the dampening of microglial activation operates likely by an inhibitory effect of E + Pr on the LPS-activated NFκB signaling pathway and the product of its target genes; *TNF-α* and *COX-2*, (3) TLR4-mediated brain inflammation reduced the survival of newly born neurons which were migrating through the RMS, (4) this reduction in the survival of newly born neurons was partially reverted by an HRT regimen containing both E and Pr.

While brain inflammation is associated with enhanced reactive astrocytes and recruitment of peripheral macrophages (Ransohoff and Brown, 2012), microglia are considered as the main target of LPS as these glial cells specifically express of TLR4 (Lehnardt et al., 2002, 2003; reviewed in Lehnardt, 2010), are rapidly activated during the acute phase of the central nervous system before the recruitment of peripheral macrophages (Greenhalgh and David, 2014) and forms the main source of inflammatory cytokines such as TNF-α and IL-6 (Lee et al., 1993; Lafortune et al., 1996; Hanisch, 2002). Thus, it is likely that the inflammatory processes observed in the present study (3 days post LPS injection) largely reflect microglial activation and its contribution to the synthesis of inflammatory cytokines (for review see Trotta et al., 2014).

### OVARIAN HORMONES AND BRAIN INFLAMMATION

*In vivo* studies support the anti-inflammatory effect of 17β-estradiol in several neurodegenerative diseases (Vegeto et al., 2006, 2008). However, relatively recent studies strongly suggest that 17β-estradiol promotes proinflammatory response to bacterial LPS by enhancing the expression of such proinflammatory genes as *TNF-α* and *IL-1β* (Calippe et al., 2008, 2010) likely by enhancing the microglial expression of TLR4 (Loram et al., 2012). The present study shows that 17β-estradiol has no significant effect on LPS-activated microglia in OVX rats. It also does not affect the proinflammatory response to LPS as assessed by the activity of the NFκB signaling pathway and the levels of TNF-α produced at the site of brain inflammation. The lack of a 17β-estradiol effect on several key elements of brain inflammation is in line with our previous observation in which an HRT based on 17β-estradiol alone did not blunt neuroimmune responses to systemically injected LPS. In contrast, an HRT consisting of 17β-estradiol and progesterone was able to dampen LPS-induced fever and LPS-activated COX-2 expression in fever controlling area of the hypothalamus (Mouihate and Pittman, 2003). Similar to TNF-α, COX-2 is also under the control of LPS-activated NFκB signaling pathway (Mouihate et al., 2005; Wu, 2005). Thus, it seems that the dampening effect of the combination of hormonal treatment with 17β-estradiol and progesterone on the brain immune response operates in similar fashion regardless of the route of the immune challenge.

While 17β-estradiol alone did not significantly affect markers of brain inflammation such as microglial activation, NFκB signaling pathway and the levels of TNF-α, we have noted that this hormone invariably enhanced the expression levels of COX-2. Owing to the important role of COX-2 in the formation of such proinflammatory prostaglandin as PGE_2_ (Rivest, 2010), it is possible that 17β-estradiol has the potential to exacerbate PGE_2_ mediated brain inflammation. It is noteworthy that activation of COX-2 can also lead to synthesis of prostaglandins endowed with anti-inflammatory properties such as PGD_2_ and its derivative PGJ_2_ (Gilroy et al., 1999; Petrova et al., 1999; Mouihate et al., 2004). The synthesis of such anti-inflammatory prostaglandins is unlikely as LPS-induced activation of microglia and the NFκB signaling were not affected by 17β-estradiol.

Collectively, our results do not support the idea that hormonal treatment based on 17β-estradiol alone is neuroprotective, at least in this brain inflammation model. It is noteworthy that 17β-estradiol has been shown to be either ineffective or exacerbates brain damage in other types of brain insults such as ischemic or hemorrhagic strokes (Harukuni et al., 2001; Carswell et al., 2004; Bingham et al., 2005; Gordon et al., 2005; Theodorsson and Theodorsson, 2005; Yong et al., 2005; De Butte-Smith et al., 2007; Nguyen et al., 2008).

**Figure A1:**
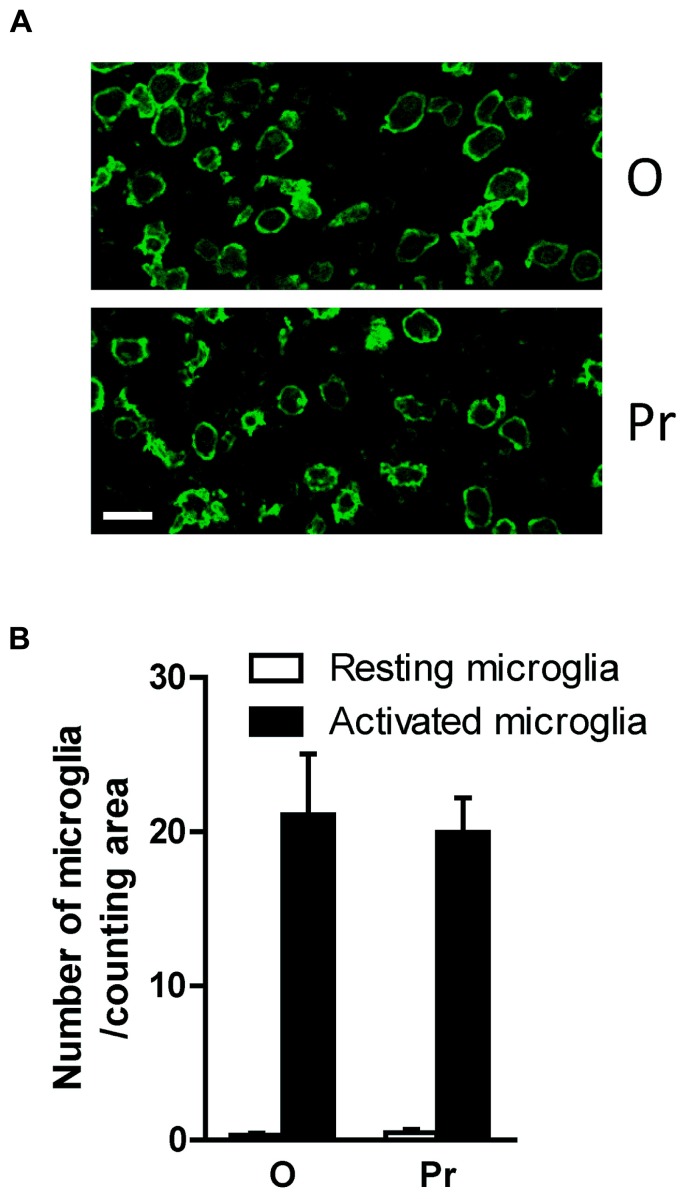
**HRT based on progesterone only does not affect LPS-induced microglial activation.** Brain inflammation was induced by intracerebral injection of LPS to OVX rats given either vehicle (O) or progesterone (Pr) treatment. Intracerebral injection of LPS to vehicle treated rats induces an activation of microglial cells expressing Iba1 (micrograph in **A**). These microglial cells show a round shape with small processes. Injection of progesterone (Pr) to OVX rats did not affect LPS-induced microglial activation (micrograph in **A**). HRT consisting of progesterone (Pr) did not significantly affect LPS-induced microglial activation (bar graph in **B**) when compared to the LPS-induced microglial activation levels seen in rats injected with oil [O (*n* = 5) *vs*. Pr (*n* = 5), *p* > 0.05]. Scale bar: 20 μm.

In a series of preliminary data, a group of OVX rats were given progesterone alone. Progesterone treatment did not elicit any significant anti-inflammatory response within the brains of OVX rats given intra-cerebral LPS (see **Figure A1** in Appendix) probably because progesterone effect is more apparent when the OVX rats are primed with 17β-estradiol. Indeed, 17β-estradiol administration increases the expression of progesterone receptors within female rat brains (Simerly et al., 1996; Scott et al., 2002; Quadros and Wagner, 2008).

### DIFFERENTIAL EFFECT ON TNF-α AND IL-6

The inhibitory effect of combinatory HRT on TNF-α production was not extended to IL-6. This observation is very peculiar as both *TNF-α* and* IL-6* genes are under the control of NFκB signaling pathway, the activity of which was significantly depressed. These data are akin to our *in vivo* studies and other’s *in vitro* observations where a phytoestrogen (resveratrol compound) inhibited LPS activated production of TNF-α but not that of IL-6 (Richard et al., 2005; Mouihate et al., 2006). The mechanism underlying this selective inhibition of TNF-α is not clear yet. It is possible that LPS activated IL-6 is mediated through activation of transcription factors other than NFκB. These transcriptional factors, which include ERK1/2, p38 MAPKs, and NF-IL6 were probably not affected by the combined HRT (Matsusaka et al., 1993; Zhang et al., 1994; Rego et al., 2011). In some circumstances, 17β-estradiol alone or in combination with progesterone has been shown to stimulate IL-6 production (Verthelyi, 2001; Brooks-Asplund et al., 2002; Isse et al., 2010), adding more complexity to the mechanism through which *IL-6* gene is affected by ovarian hormones.

### OVARIAN HORMONES AND NEUROGENESIS

Brain inflammation hampers neurogenesis (Monje et al., 2003) likely via microglia derived TNF-α (Lafortune et al., 1996; Hanisch, 2002; Lambertsen et al., 2009; Nimmervoll et al., 2013). In the present study, we confirmed that the brain inflammatory response, as illustrated by microglial activation and TNF-α protein expression, resulted in decreased number of newly born neurons. This observation is in line with previous studies demonstrating the deleterious effect of TNF-α on the survival of neural precursor cells (Ekdahl et al., 2003; Iosif et al., 2006; Keohane et al., 2010; Ekdahl, 2012). More interestingly, we demonstrated for the first time that an HRT regimen containing both 17β-estradiol and progesterone, not only blunted brain inflammation but it also dampened brain inflammation-induced reduction in newly born neurons. Such effect was absent when progesterone was omitted from the HRT regimen.

However, ovarian hormones can also affect neurogenesis through a sensitization/desensitization to LPS effects. Indeed, TLR4 receptors are expressed on neural stem cells and play a major role in neurogenesis (Rolls et al., 2007; Shechter et al., 2008). There are indications that ovarian hormones can affect the expression levels of TLR4 in immune competent cells. For example, 17β-estradiol enhances TLR4 expression in macrophages (Rettew et al., 2009), while progesterone depresses its expression in the brain of mice with experimental autoimmune encephalomyelitis (Garay et al., 2012) or that of rats subjected to subarachnoid hemorrhage (Wang et al., 2011). Whether ovarian hormones alter the expression of TLR4 on neural precursor cells and thus prime these newly born cells to the deleterious effect of LPS is still an open question.

## CONCLUSION

LPS-induced brain inflammation resulted in activated microglial cells and enhanced levels of molecular markers of inflammation such as NFκB signaling pathway and its proinflammatory target proteins (TNF-α and COX-2). HRT based on 17β-estradiol alone was devoid of anti-inflammatory properties in TLR4-induced brain inflammation. In contrast, both LPS-activated microglia and the resulting activated molecular proinflammatory machinery were significantly reduced in OVX rats given an HRT regimen containing 17β-estradiol and progesterone. Interestingly, the anti-inflammatory effect of complete HRT created conducive environment for the survival of newly born neurons.

## AUTHOR CONTRIBUTION

Abdeslam Mouihate designed the research, performed research, analyzed data, and wrote the manuscript.

## Conflict of Interest Statement

The author declares that the research was conducted in the absence of any commercial or financial relationships that could be construed as a potential conflict of interest.
